# Brain dopamine receptor system is not altered in obesity: Bayesian and frequentist meta‐analyses

**DOI:** 10.1002/hbm.26534

**Published:** 2023-11-11

**Authors:** Kyoungjune Pak, Lauri Nummenmaa

**Affiliations:** ^1^ Department of Nuclear Medicine and Biomedical Research Institute Pusan National University Hospital Busan Republic of Korea; ^2^ School of Medicine Pusan National University Busan Republic of Korea; ^3^ Turku PET Centre University of Turku Turku Finland; ^4^ Turku PET Centre Turku University Hospital Turku Finland; ^5^ Department of Psychology University of Turku Turku Finland

**Keywords:** dopamine receptor, obesity, positron emission tomography

## Abstract

Feeding induces dopamine release in the striatum, and a dysfunction of the dopaminergic reward system can lead to overeating, and obesity. Studies have reported inconsistent findings of dopamine receptor (DR) positron emission tomography scans in obesity. Here we investigated the association between DR availability and overweight/obesity using Bayesian and frequentist meta‐analysis. We performed a systematic search of Embase, Medline, Scopus and Web of Science for studies that compared striatal DR availability between lean subjects and overweight/obese subjects. The standardized mean difference (Hedge's g) of DR availability was calculated after extraction of data from each study. Studies were divided into two groups according to the definition of overweight/obese subjects (body mass index [BMI] cutoff of 25 and 30 kg/m^2^). Both Bayesian and frequentist meta‐analysis was done in R Statistical Software version 4.2.2 (The R Foundation for Statistical Computing). Nine studies were eligible for inclusion in this study. Three studies with C11‐raclopride, one with C11‐PNHO, two with F18‐fallypride, one with I123‐IBZM, one with C11‐NMB and one with both C11‐raclopride and C11‐PNHO were included. In Bayesian meta‐analysis, the standardized mean difference of DR availability between lean and overweight/obese subjects markedly overlapped with zero regardless of BMI cutoff for obesity. In frequentist meta‐analysis, the pooled standardized mean difference of DR availability did not show the significant difference between lean and overweight/obese subjects. There was an effect of the radiopharmaceutical on the standardized mean difference of DR availability in meta‐analysis of BMI cutoff of 25 kg/m^2^. In conclusion, brain DR availability is not different between lean and overweight/obese subjects. However, the effect is dependent on the radiopharmaceutical and the degree of obesity. Further studies with multi‐radiopharmaceutical in the same individuals are needed to understand the association between DR and obesity.

## Graphical abstract

- Brain dopamine receptor availability is not different between lean and overweight/obese subjects according to both Bayesian and frequentist meta‐analyses.
- However, the effect is dependent on the radiopharmaceutical and the degree of obesity.

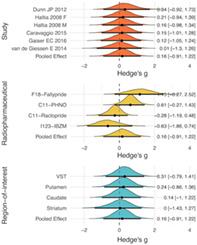

## 1 INTRODUCTION

Obesity has nearly tripled worldwide since 1975 and has become one of the major public health threats. Obesity is a risk factor for malignancies of the colon (Na & Myung, 2012), pancreas (Gukovsky et al., 2013), thyroid (Mijovic et al., 2011), liver (Alzahrani et al., 2014), and uterus (Gu et al., 2013) as well as for cardiovascular disease and diabetes mellitus (Burke et al., 2008).

Obesity is caused by an imbalance between energy intake and expenditure over a long period of time (Morton et al., 2014). The brain plays a critical role in controlling energy balance (Morton et al., 2014). Feeding induces dopamine release in the striatum (Small et al., 2003), and a dysfunction of the dopaminergic reward system can lead to overeating, and obesity (Volkow et al., 2011). However, there is no direct method to measure the synaptic dopamine concentration in the human brain. Therefore, positron emission tomography (PET) and single‐photon emission computed tomography (SPECT) using dopamine receptor (DR) radiopharmaceuticals has been adopted to understand the role of dopaminergic system in obesity. A landmark study by Wang et al showed that DR availability was lower in severely obese subjects (the mean body mass index [BMI] of 51.2 kg/m^2^) than in lean subjects (Wang et al., 2001). After that, several studies reported inconsistent findings of DR PET scans in obesity; higher (Gaiser et al., 2016) or not different (Eisenstein et al., 2013) DR availability in obese subjects compared to lean subjects. In addition, some researchers suggested non‐linear relationship between DR availability and BMI (Janssen & Horstmann, 2022; van Galen et al., 2018). However, previous studies included the small sample size, the discrepancies in region‐of‐interest (ROI) and the variety of radiopharmaceuticals, leading to these inconsistent findings. Therefore, it is more timely than ever to meta‐analyze the previous studies of association between DR availability measured from PET or SPECT and obesity. In this study, we divided the previous publications into two groups according to the definition of overweight/obese subjects (BMI cutoff of 25 and 30 kg/m^2^) and we investigated the association between DR availability and overweight/obesity with frequentist as well as Bayesian meta‐analysis.

## 2 MATERIALS AND METHODS

### 2.1 Data search and study selection

We performed a systematic search of Embase, Medline, Scopus and Web of Science from inception to November 2022 for articles published in English using the keywords “dopamine receptor,” “obesity” and “positron emission tomography OR single‐photon emission computed tomography.” All searches were limited to human studies. The inclusion criteria were neuroimaging studies which (1) compared DR availability of lean subjects and that of overweight/obese subjects and (2) measured DR availability within the striatum (whole striatum, caudate nucleus, putamen, nucleus accumbens, ventral striatum) using PET or SPECT scans. Reviews, abstracts, and editorial materials were excluded. Further, duplicate articles were excluded. If there was more than one study using the same set of patients, the study reporting information most relevant (i.e., initial patient‐control differences) to the present study was included. Two authors performed the literature search and screened the articles independently, and discrepancies were resolved by consensus.

### 2.2 Data extraction and statistical analysis

**FIGURE 1:**
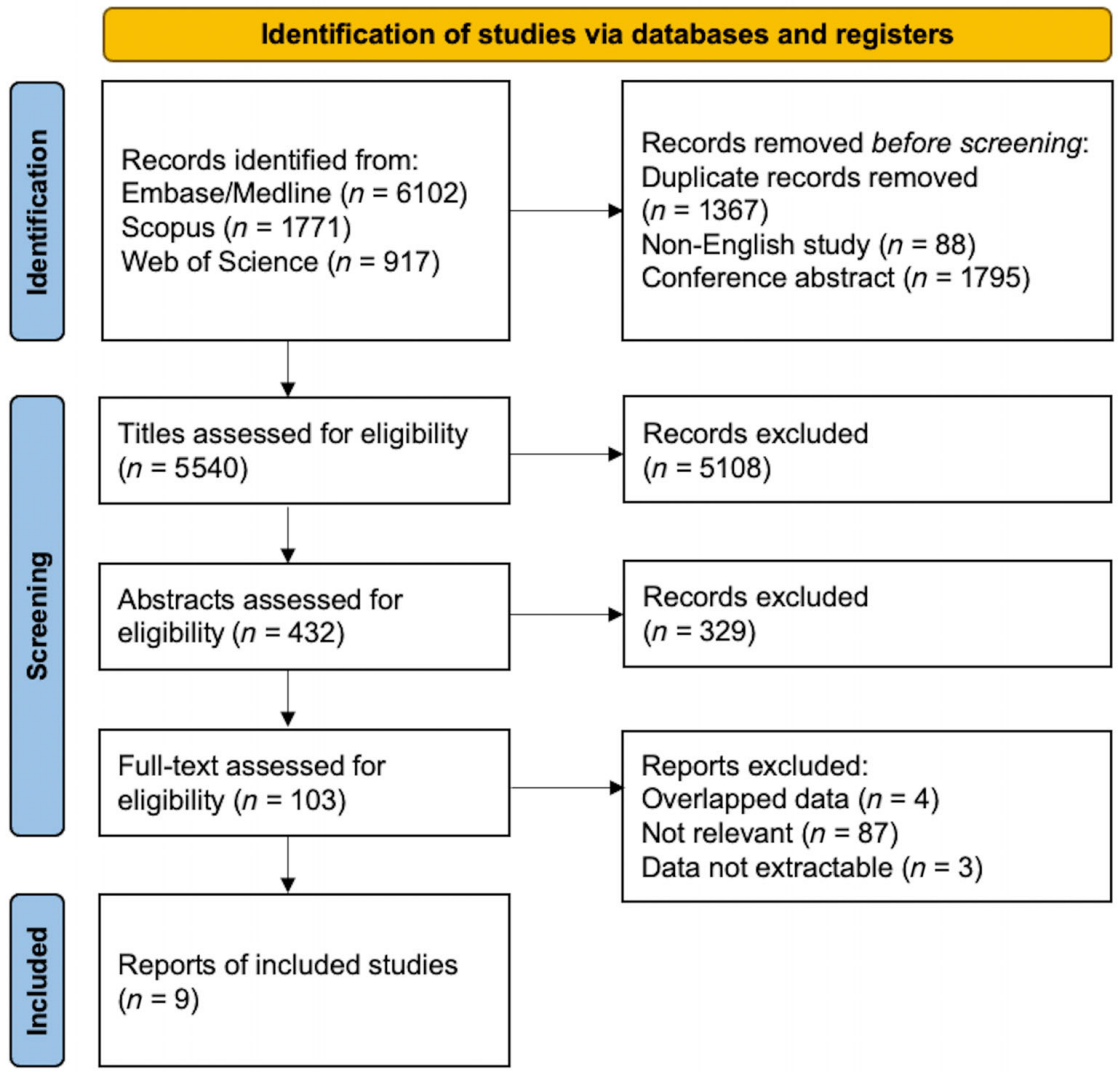
Flowchart for literature searches and data extraction.

Two reviewers independently extracted the following information from the reports: First author, year of publication, country, radiopharmaceuticals, the mean BMI of lean subjects and overweight/obese subjects, and ROI. First, we extracted the mean and the standard deviation of DR availability and the number of subjects in each group, directly from each study, if provided by the authors. If they were not provided by the authors, BMI and DR availability were extracted from the figures using WebPlotDigitizer version 4.6 (https://automeris.io/WebPlotDigitizer/): Figure 1 from the study by Caravaggio et al. (2015) and supplementary figure 5 from the study by Dunn et al. (2012). Original subject‐wise data matrix was used for one study (Tuominen et al., 2015). Subsequently, the standardized mean difference (Hedge's g) of DR availability was calculated for each study.

The standardized mean difference of DR availability between lean and overweight/obese subjects were first investigated using Bayesian hierarchical modeling with brms (Bürkner, 2017, 2018, 2021) that applies the Markov‐Chain Monte Carlo sampling tools of RStan (Stan Development Team, 2022). We set up a model with the standardized mean difference of DR availability as a dependent variable and added study, radiopharmaceutical and ROI as random intercepts to allow the standardized mean difference of DR availability to vary between studies, radiopharmaceuticals and ROIs. Bayesian models were estimated using four Markov chains, each of which had 4000 iterations including 1000 warm‐ups, thus totaling 12,000 post‐warmup samples. The sampling parameters were slightly modified to facilitate convergence (max treedepth = 30). Also, complementary frequentist meta‐analysis was performed using metafor (Viechtbauer, 2010). Heterogeneity among studies was assessed using Cochran's *Q* and *I* ^2^ statistics, as described previously (Higgins et al., 2003). The pooled effect size was estimated using random‐effects model. Meta‐regression was performed to explore potential sources of heterogeneity due to radiopharmaceutical and the mean BMI of overweight/obese subjects. Statistical analysis was carried out in R Statistical Software version 4.2.2 (The R Foundation for Statistical Computing).

## 3 RESULTS

### 3.1 Study characteristics

The search identified 6102 articles from Embase/Medline, 1771 from Scopus, 917 from Web of Science. After excluding duplicate records (*n* = 1367), conference abstracts (*n* = 1795) and non‐English publications (*n* = 88), studies were assessed for eligibility after screening the title or abstract. After reviewing the full text of 103 articles, nine studies were eligible for inclusion in this study (Caravaggio et al., 2015; Dunn et al., 2012; Eisenstein et al., 2013; Gaiser et al., 2016; Guo et al., 2014; Haltia et al., 2008; Tuominen et al., 2015; van de Giessen et al., 2014; Wang et al., 2001) (Figure 1). Three studies with C11‐raclopride (Haltia et al., 2008; Tuominen et al., 2015; Wang et al., 2001), one with C11‐PNHO (Gaiser et al., 2016), two with F18‐fallypride (Dunn et al., 2012; Guo et al., 2014), one with I123‐IBZM (van de Giessen et al., 2014), one with C11‐NMB (Eisenstein et al., 2013) and one with both C11‐raclopride and C11‐PNHO (Caravaggio et al., 2015) were included. The definition of lean and overweight/obese subjects varied across the studies. In four studies, subjects were divided into two groups according to BMI cutoff of 30 kg/m^2^ (Eisenstein et al., 2013; Guo et al., 2014; Tuominen et al., 2015; Wang et al., 2001). Three studies that defined lean subjects as BMI less than 25 kg/m^2^ and obese subjects as BMI more than 30 (Dunn et al., 2012; Gaiser et al., 2016) or 35 kg/m^2^ (Tuominen et al., 2015) were included in both analyses of studies with BMI cutoff of 25 and 30 kg/m^2^. In one study, lean subjects were defined as BMI less than 24 kg/m^2^ and overweight subjects as BMI more than 27 kg/m^2^ (Haltia et al., 2008). As the study by Caravaggio et al., included subjects of BMI between 18.6 and 27.8 kg/m^2^, we divided subjects into two groups with BMI cutoff of 25 kg/m^2^ (Caravaggio et al., 2015). Due to the heterogeneity in the definition of obesity, we ran the meta‐analyses separately with datasets where obesity was defined as BMI > 25 or BMI > 30 kg/m^2^. The study characteristics are summarized in Table 1.

**TABLE 1:** The study characteristics.

Study		Radiopharmaceutical	Region‐of‐interest	BMI cutoff (kg/m^2^)	Lean subjects			Overweight/obese subjects		
					No.	BMI (kg/m^2^)		No.	BMI (kg/m^2^)	
						Mean	Range		Mean	Range
Caravaggio et al. (2015)		C11‐PHNO	VST	25	16	21.6	18.6 ~ 24.9	10	26.6	25 ~ 27.8
		C11‐Raclopride	VST	25	24	21.7	18.6 ~ 24.9	11	26.6	25 ~ 27.8
Dunn et al. (2012)		F18‐Fallypride	Caudate	25/30	8	23	~24.9	14	40	30~
Eisenstein et al. (2013)		C11‐NMB	Caudate/Putamen/VST/Striatum	30	15	22.6	18.9 ~ 27.7	15	40.3	33.2 ~ 47
Gaiser et al. (2016)		C11‐PHNO	Caudate/Putamen/VST	25/30	14	22.3	18.5 ~ 24.9	14	35.3	30~
Guo et al. (2014)		F18‐Fallypride	Caudate/Putamen/VST	30	23	22.4	~30	20	36.1	30~
Haltia et al. (2008)	Female	C11‐Raclopride	Putamen/VST	25	6	21.4	~24	6	34.4	27~
	Male	C11‐Raclopride	Putamen/VST	25	6	21.9	~24	6	31.8	27~
Tuominen et al. (2015)		C11‐Raclopride	Caudate/Putamen/VST	30	20	22.3	~30	25	41.3	35~
van de Giessen et al. (2014)		I123‐IBZM	Striatum	25/30	15	21.8	18.5 ~ 24.9	15	42.9	36.3 ~ 56.5
Wang et al. (2001)		C11‐Raclopride	Striatum	30	10	24.7	21 ~ 28	10	51.2	42 ~ 60

Abbreviations: BMI, body mass index; VST, ventral striatum.

### 3.2 Brain dopamine receptor and overweight/obesity: A Bayesian meta‐analysis

**FIGURE 2:**
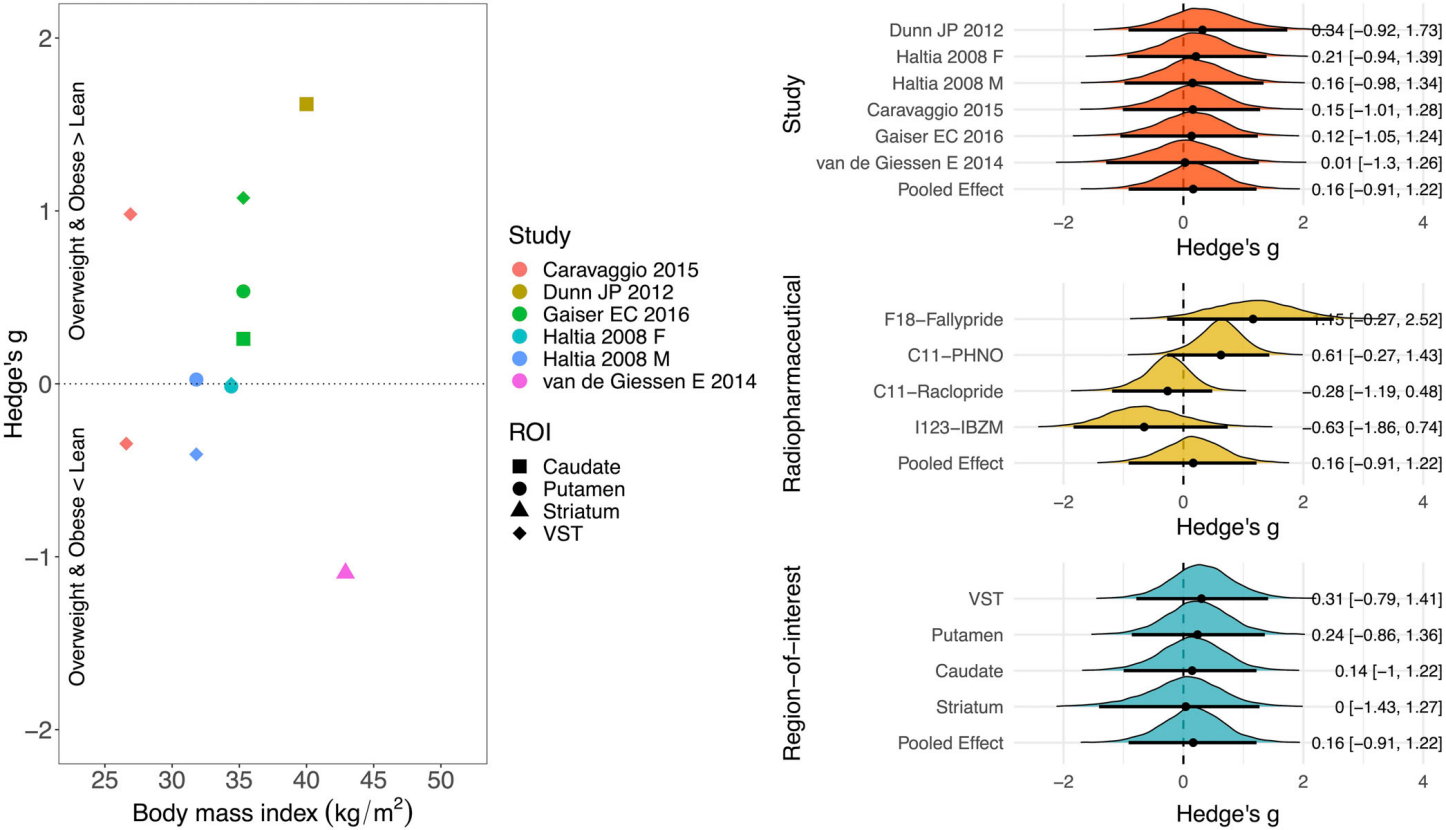
Studies of body mass index (BMI) cutoff of 25 kg/m^2^, left: scatter plot of the standardized mean difference of dopamine receptor (DR) availability between lean and overweight/obese subjects and BMI, right; posterior probability distributions with their median (point), 80% (thick line) and 95% (thin line) posterior intervals, describing the standardized mean difference of DR availability. Posterior located on the positive side of the zero line suggests higher DR availability in overweight/obese subjects. ROI, region‐of‐interest; VST, ventral striatum.

**FIGURE 3:**
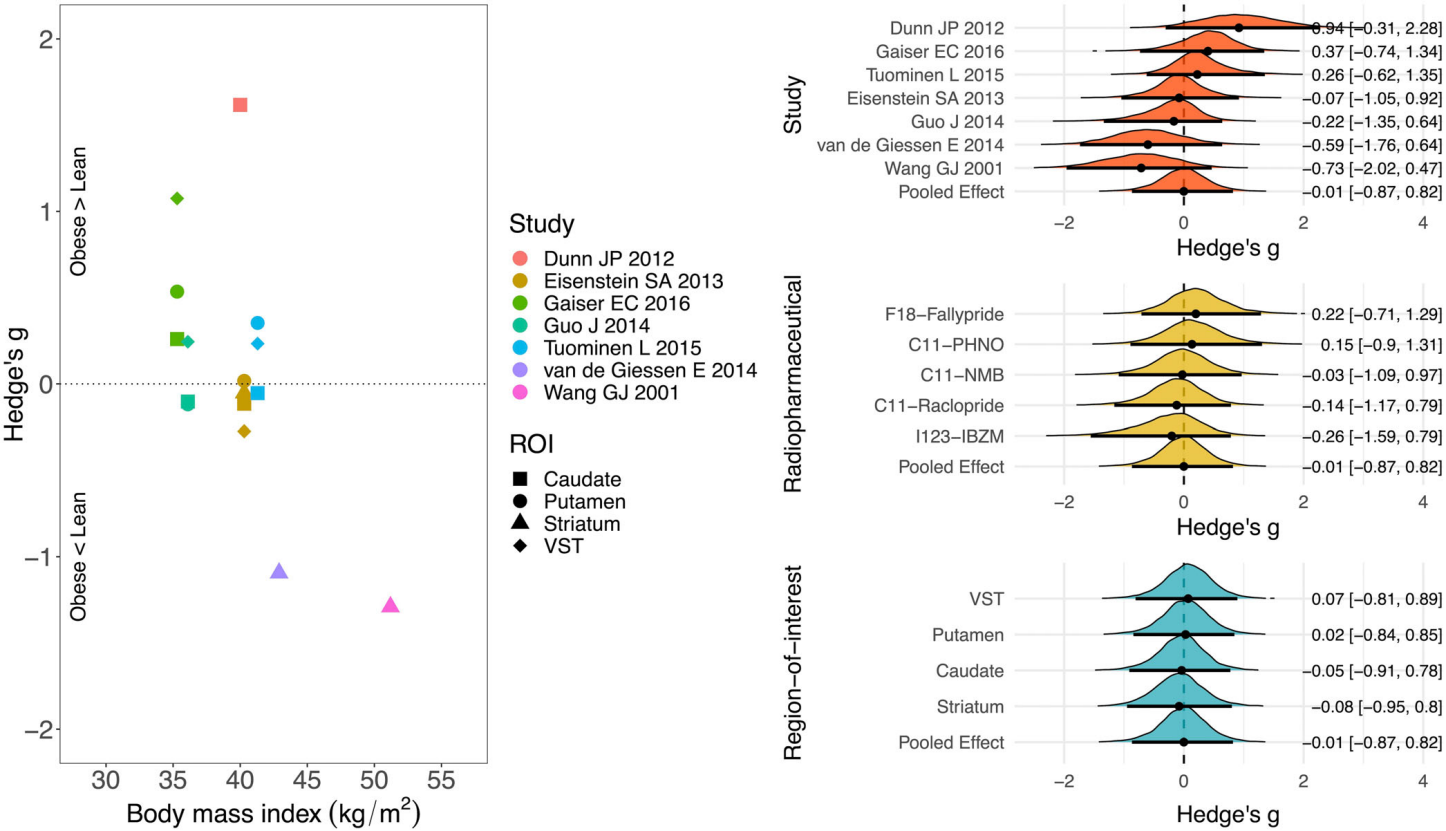
Studies of body mass index (BMI) cutoff of 30 kg/m^2^, left: scatter plot of the standardized mean difference of dopamine receptor (DR) availability between lean and obese subjects and BMI, right: posterior probability distributions with their median (point), 80% (thick line) and 95% (thin line) posterior intervals, describing the standardized mean difference of DR availability. Posterior located on the positive side of the zero line suggests higher DR availability in obese subjects. ROI, region‐of‐interest; VST, ventral striatum.

The distribution of the standardized mean difference of DR availability and mean BMI of overweight/obese subjects are shown in Figure 2 (BMI cutoff for overweight/obesity 25 kg/m^2^) and Figure 3 (BMI cutoff for obesity 30 kg/m^2^). In the analysis of BMI cutoff of 25 kg/m^2^, the standardized mean difference of DR availability between lean and obese subjects overlapped with zero. There was a more support for the association with radiopharmaceutical that overweight/obese subjects in studies with F18‐Fallypride and C11‐PHNO has higher DR availability and those in studies with C11‐Raclopride and I123‐IBZM has lower DR availability than lean subjects (Figure 2). In the analysis of BMI cutoff of 30 kg/m^2^, the standardized mean difference of DR availability between lean and overweight/obese subjects markedly overlapped with zero (Figure 3).

### 3.3 Brain dopamine receptor and overweight/obesity: Frequentist meta‐analysis

**FIGURE 4:**
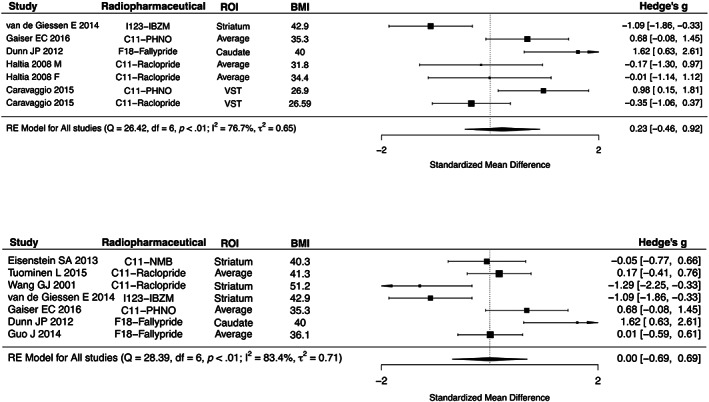
Forest plots of studies of body mass index (BMI) cutoff of 25 and 30 kg/m^2^. ROI, region‐of‐interest; VST, ventral striatum.

A single ROI from each study was included in frequentist meta‐analysis. If the study reported results for more than one ROI (Gaiser et al., 2016; Guo et al., 2014; Haltia et al., 2008; Tuominen et al., 2015), mean DR availability across striatal subregions was calculated. In the meta‐analysis of studies with BMI cutoff of 25 kg/m^2^, the pooled standardized mean difference of DR availability did not show the significant difference between lean and overweight/obese subjects (0.23, 95% confidence interval −0.46 ~ 0.92, *I* ^2^ = 76.7%). Meta‐regression analyses revealed a statistically significant effect of radiopharmaceutical on the pooled effect size (C11‐Raclopride, −1.0504, −1.8265 ~ −0.2742, *p* = .0080; I123‐IBZM, −1.9141, −2.8656 ~ −0.9626, *p* < .0001). In a meta‐analysis of studies with BMI cutoff of 30 kg/m^2^, the pooled standardized mean difference of DR availability was not significantly different between lean and overweight/obese subjects (0, −0.69 ~ 0.69, *I* ^2^ = 83.4%). Meta‐regression analyses revealed that the mean BMI of overweight/obese subjects is negatively associated with the standardized mean difference of DR availability (−0.1214, −0.2397 ~ −0.0031, *p* = .0442) (Figure 4).

## 4 DISCUSSION

Our main finding was that brain DR availability does not differ between lean and overweight/obese subjects. This result was confirmed in both Bayesian and frequentist meta‐analyses and with two different criteria (BMI > 25 kg/m^2^ or BMI > 30 kg/m^2^) for overweight/obesity. However, the effect sized varied as the function of the used radiopharmaceuticals and the degree of obesity. Altogether these results suggest that striatal downregulation of D2R is not a common feature of obesity despite the centrality of dopaminergic system in feeding and reward (Bello & Hajnal, 2010).

Obesity is caused by an imbalance between the energy intake and expenditure (Bellisle et al., 2012). Feeding is controlled by both homeostatic regulatory brain circuits and those involved in reward and motivation (Volkow et al., 2013). Dopamine is one of the neurotransmitters involved with eating behavior through modulation of the rewarding properties of food and the motivation and desire for food consumption (Bello & Hajnal, 2010). There are two major hypotheses regarding the role of dopamine in obesity. The first hypothesis, dopamine hyperresponsiveness, proposes that there is a hypersensitivity to rewards and increased behavioral salience toward food, resulting in the excessive intake of palatable foods (Kessler et al., 2014; Verbeken et al., 2012). The second hypothesis, reward deficit model, states that subjects who are insensitive to rewards overeat to increase their endogenous dopamine levels to a normal amount of pleasure (Kessler et al., 2014; Verbeken et al., 2012). However, it is not possible to measure the synaptic dopamine levels directly in the human brain. Therefore, PET with DR radiopharmaceuticals has been used to understand the role of dopaminergic system in obesity. However, it is difficult to interpret the relationships between DR availability measured from PET scans and the synaptic dopamine level. Low DR binding potential could represent (1) low density of existing DR, (2) low affinity to bind DR, or (3) greater amount of endogenous dopamine which competes with DR radiopharmaceutical to bind DR (Guo et al., 2014; Janssen & Horstmann, 2022).

Nine such studies were included in this meta‐analysis. There was no significant difference of DR availability between lean and overweight/obese subjects in four studies in any striatal ROIs (Eisenstein et al., 2013; Guo et al., 2014; Haltia et al., 2008; Tuominen et al., 2015), in one study no effect in caudate and putamen (Gaiser et al., 2016) and also in one study with C11‐Raclopride, there was no significant correlation between BMI and DR availability (Caravaggio et al., 2015). In the study by Dunn et al., obese subjects had higher DR availability in caudate (Dunn et al., 2012) and in the study by Caravaggio et al., with C11‐PHNO, there was a positive correlation between BMI and DR availability (Caravaggio et al., 2015). In two studies, obese subjects had lower DR availability than lean subjects (van de Giessen et al., 2014; Volkow et al., 2013).

Because the studies had different definition of overweight/obese subjects, we conducted two separate meta‐analyses with studies using two different cutoffs for obesity (BMI > 25 kg/m^2^ and BMI > 30 kg/m^2^). Neither meta‐analysis provided support for overall obesity‐dependent modulation of striatal DR. In the meta‐analysis of BMI cutoff of 25 kg/m^2^, five studies were included. The study by Caravaggio et al., included overweight subjects of BMI between 25 and 27.8 kg/m^2^ without obese subjects (Caravaggio et al., 2015) and the study by Haltia et al., included both overweight and obese subjects from BMI 27 kg/m^2^ (Haltia et al., 2008). The other three studies were included in both analysis of BMI cutoff of 25 and 30 kg/m^2^ (Dunn et al., 2012; Gaiser et al., 2016; van de Giessen et al., 2014). In addition to the inconsistency of BMI cutoff of overweight/obese subjects, the minimum BMI of obese subjects in each study ranged from 30 to 42 kg/m^2^ even with the studies of the same BMI cutoff of 30 kg/m^2^. Therefore, the mean BMI of lean subjects as well as the mean BMI of obese subjects varied widely across the studies. Also, the heterogeneity of sex and age of participants in each study could affect the findings of this study. Although there was no difference between lean and overweight/obese subjects in DR availability, there was an effect of radiopharmaceutical on both Bayesian and frequentist meta‐analysis of BMI cutoff of 25 kg/m^2^. One study with F18‐Fallypride (Dunn et al., 2012) and two studies with C11‐PHNO showed the higher DR availability in overweight/obese subjects while two studies with C11‐Raclopride (Caravaggio et al., 2015; Haltia et al., 2008) and one study with I123‐IBZM (van de Giessen et al., 2014) did not show the difference of DR availability, which might elucidate this effect of radiopharmaceutical on the result.

In a meta‐analysis with BMI cutoff of 30 kg/m^2^, in addition to three studies that included in both analysis of BMI cutoff of 25 and 30 kg/m^2^, four additional studies with obese subjects were included. Similar with the result of BMI cutoff of 25 kg/m^2^, there was no difference between lean and overweight/obese subjects in DR availability. However, meta‐regression showed that the mean BMI of overweight/obese subjects was negatively associated with the standardized mean difference of DR availability. The subjects in the studies with the two different BMI cutoffs also varied with respect to the degree of the severity of obesity: The mean BMI of overweight/obese subjects in a meta‐analysis of BMI cutoff of 30 kg/m^2^ was higher than that of 25 kg/m^2^ (mean BMI of 40.1 vs. 34.9 kg/m^2^). Also, the study by Wang et al. (2001) with the highest mean BMI of obese subjects (mean BMI of 51.2 kg/m^2^) was included in a meta‐analysis of BMI cutoff of 30 kg/m^2^. The meta‐analysis with BMI cutoff of 30 kg/m^2^ thus also includes obese subjects with the wider range of BMI.

Against our expectation, there was no effect of ROI on the standardized mean difference of DR availability between lean and overweight/obese subjects, although all ROIs could be included in Bayesian meta‐analysis (unlike in the frequentist meta‐analysis that included a single representative ROI from each study). Five DR radiopharmaceuticals were included in this meta‐analysis and each radiopharmaceutical has its own profile of DR affinity, competition with endogenous dopamine and agonist/antagonist. Both C11‐Raclopride and F18‐Fallypride, D2/D3 antagonists, have a similar affinity to D2 and D3 and compete with endogenous dopamine (Dunn et al., 2012; Volkow et al., 1994). I123‐IBZM, a D2/D3 antagonist for SPECT, competes with endogenous dopamine, similar with C11‐Raclopride (Janssen & Horstmann, 2022). C11‐NMB, a D2‐selective antagonist, is not replaceable by endogenous dopamine (Eisenstein et al., 2013). C11‐PHNO, a D3‐preferring agonist, is more sensitive to endogenous dopamine levels than radiopharmaceuticals of DR antagonists (Gaiser et al., 2016), therefore, considered superior to detect synaptic dopamine release (van Wieringen et al., 2014). Therefore, it is critical to consider the feature of each radiopharmaceutical while interpreting the results from PET and SPECT scans.

Previous systematic reviews investigated the association between brain DR and obesity (Janssen & Horstmann, 2022; van Galen et al., 2018). However, there are limitations in their studies. First, both studies are systematic reviews without a statistical approach of meta‐analysis (either Bayesian or Frequentist models). Therefore, there was no quantitative approach on the association of brain DR with obesity. Second, in their studies, four studies with severe obesity were included, which led to the conclusion of lower brain DR in severe obesity, however, two research groups (from NIH and Amsterdam) published four studies with overlapping subjects with I123‐IBZM and C11‐Raclopride. In our study, we excluded the overlapping studies from the same research groups and underwent the statistical approach of meta‐analysis (both Bayesian and Frequentist models) to evaluate the association of brain DR with obesity.

In this study, both Bayesian and frequentist models were used to investigate the association of brain DR with obesity. Bayesian model describes the probability of an event occurrence based on previous knowledge of the conditions associated with the event (Hackenberger, 2019). Frequentist model calculates the probability of obtaining another data set at least as extreme as the one collected (Fornacon‐Wood et al., 2022). Both models are different approaches to statistical inference, differing in the interpretation of uncertainty. Therefore, with two models together in this study, we could overcome the limitation of each model in investigation of the association of brain DR with obesity.

There are several limitations in this study. First, only a small number of studies could be included in this meta‐analysis. After a systematic search for publication, several papers were assessed for eligibility. However, most of them were published from the same institution with overlapping subjects and we selected the most relevant publication among the pool of articles with same subjects. In addition, as two different BMI cutoffs were applied in these studies, five studies with BMI cutoff of 25 kg/m^2^ and seven studies with that of 30 kg/m^2^ could be included in meta‐analysis. Also, there is a possibility that there are alterations in DR availability at higher BMI levels (>35 kg/m^2^), as there is some evidence for this in the literature (van de Giessen et al., 2014; Wang et al., 2001). Finally, five DR radiopharmaceuticals were used in studies included in this meta‐analysis and each radiopharmaceutical has its own receptor‐binding property. Therefore, we should be cautious when interpreting the findings from the studies included in this meta‐analysis.

## 5 CONCLUSIONS

Brain DR is not different between lean and overweight/obese subjects and these meta‐analytic findings from patients also align with the recent large‐scale study of non‐obese healthy subjects (Malén et al., 2022). There is an effect of the variety of radiopharmaceuticals and the degree of the severity of obesity on this result, therefore, we still cannot exclude the association of brain DR with obesity. For example, it is possible that obesity alters the threshold for endogenous dopamine release following feeding rather than the receptor densities (Small et al., 2003), or that obesity alters the molecular coupling between dopamine and other neurotransmitter systems such as endogenous opioid systems that has been consistently linked with obesity (Burghardt et al., 2015; Karlsson et al., 2016; Tuominen et al., 2015). Further studies with multi‐radiopharmaceutical in the same individuals are needed to elucidate the potential links between DR and obesity.

## AUTHOR CONTRIBUTIONS

**Kyoungjune Pak:** Conceptualization; methodology; formal analysis; software; visualization; writing. **Lauri Nummenmaa:** Conceptualization; methodology; formal analysis; software; visualization; writing.

## CONFLICT OF INTEREST STATEMENT

The authors declare no conflicts of interest.

## Data Availability

The data that support the findings of this study are available from the corresponding author upon reasonable request.
